# Effect of Pore Structure on Thermal Conductivity and Mechanical Properties of Autoclaved Aerated Concrete

**DOI:** 10.3390/ma14020339

**Published:** 2021-01-11

**Authors:** Gonglian Chen, Fenglan Li, Pengfei Jing, Jingya Geng, Zhengkai Si

**Affiliations:** 1 Department of Civil Engineering, School of Civil Engineering and Communication, Huayuan Campus, North China University of Water Resources and Electric Power, No. 36 Beihuan Road, Zhengzhou 450045, China; lifl64@ncwu.edu.cn; 2 International Joint Research Lab for Eco-Building Materials and Engineering of Henan, Huayuan Campus, North China University of Water Resources and Electric Power, No. 36 Beihuan Road, Zhengzhou 450045, China; 201510319123@stu.ncwu.edu.cn (P.J.); 2018911008@yrcti.edu.cn (J.G.); 3 Henan Xing’an New Building Materials Co., Ltd., Xingyang National Electric Industrial Park, Zhengzhou 450000, China; sizhengkai@hnxan.com

**Keywords:** autoclaved aerated concrete (AAC), thermal conductivity, pore structure, base material, mechanical properties

## Abstract

With the premise of investigating mechanical properties, the thermal conductivity of autoclaved aerated concrete (AAC) is a key index of self-insulation block walls for building energy conservation. This study focused on the effect of pore structures on the mechanical performance and thermal conductivity of AAC with the comparison of AAC base materials. Different kinds of AAC and their base materials were prepared and experimentally investigated. While maintaining a consistent mix proportion of the AAC base material, the pore structure of AAC was changed by the dosage of aluminum power/paste, foam stabilizer, and varying the stirring time of aluminum paste. The steam curing systems of AAC and the base material were determined based on SEM (Scanning Electronic Microscopy) and XRD (X-ray Diffraction) tests. With almost the same apparent density, the pore size decreased with the increasing content of foam stabilizer, and the mixing time of aluminum paste and foam stabilizer has a great influence on pore size. The thermal conductivity test and compressive test results indicated that that pore size had an effect on the thermal conductivity, but it had little effect on the compressive strength, and the thermal conductivity of sand aeration AAC was 8.3% higher than that of fly ash aeration AAC; the compressive strength was 10.4% higher, too. With almost the same apparent density, the regression mathematical model indicates that the thermal conductivity of AAC increased gradually with the increase of pore size, but it had little effect on the compressive strength. From the test results of basic mechanical properties, the mechanical model of cubic compressive strength, elastic modulus, axial compressive strength, and splitting tensile strength was obtained. The proposed stress–strain relationship model could well describe the relationship of AAC and the base material at the rising section of the curve.

## 1. Introduction

As an energy-saving building material with good performances of lightweight and thermal insulation, autoclaved aerated concrete (AAC) has been widely used in building structures as the filling walls or the load-bearing walls [1,2,3,4]. With a premise of required strength and density, AAC should be produced with lowest thermal conductivity to be used as the enveloped self-insulation block walls [4,5,6,7].

AAC is a kind of two-phase porous material composed of continuous solid phase (cement matrix) and discrete gas phase (air pore). The pores in aerated concrete are composed of macro pores and micro pores. The macro pores are formed by the gas overflow after the addition of the gas generation agent; the shape is spherical or nearly spherical, and the pore diameter is basically among 0.5–2 mm. In the range of a few tenths of a millimeter to a few millimeters, the micro pores exist in the wall of the macro pores. The porosity of AAC is generally 70–80%, in which the porosity caused by hydrogen generated by the chemical reaction of aluminum paste in alkali solution accounts for about 45–55%, which is macro porosity; the capillary left by water evaporation accounts for about 20–40%. For AAC, the macro pores are bigger and there are many more than the ordinary concrete, which has a great influence on the strength and thermal conductivity of concrete [8,9].

It is known that the thermal performance of AAC relates to the porosity: the larger the porosity, the lower the thermal conductivity, which is greatly affected by the addition of an Al component, because the air pore is mainly made of aluminum powder [7,8,9,10,11,12,13,14]. By using Al powder, Al paste and highly dispersed Al particles, different pore shapes of AAC can be generated. Some researchers used industrial waste instead of aluminum powder as a gas generation agent [15,16], which was aluminum waste or municipal solid waste incineration bottom ash. The gas generation mechanism was similar to aluminum powder, but the AAC density was relatively big, which was just as an exploratory study of aluminum powder replacement. Esmaily [17] used alkali-activated slag in the place of usual cementitious materials in the production of AAC. The substitution altered the autoclave curing stage by steam curing in the AAC production process, and since the AAC density was big, the thermal conductivity would be large. However, with the same volume density, no research has examined the effect of aeration methods on the pore shape of AAC nor the effect of pore structure on the thermal conductivity of AAC. Except the mathematical analysis made by Li [18] to investigate the effect of pore form on the thermal conductivity of AAC, less attention was paid to the effect of pore structure characterized by diameter, size, and distribution [19], which limits the technical development of AAC with ideal thermal insulation.

To intensively study the forming mechanisms of the pores and the heat transfer characteristics of AAC, researches on the base material is valuable, which consists of the hydration products made of the same mix proportion of AAC without the addition of an aerating agent. The base materials and AAC with different dosages of cement, lime, water, and sand were experimentally studied without considering the autoclaved curing in the manufacturing process by Richard [20]. Chalermphan [21] presented the base material mixes cured in water and air for 3, 7, and 28 days. Chen [22] has pointed out that high steam pressure curing can significantly reduce the reaction time and obtain products with high compressive strength. Isu [23] pointed out that the content of quartz in sand was directly proportional to the strength of AAC due to the higher reaction of quartz with calcium oxide.

At present, the research results showed that the porosity has a great influence on the strength of AAC, but the influence of pore size and pore shape on the strength is generally not considered. For ordinary concrete, some researchers have also studied the influence of pore size distribution and pore morphology on the strength [24,25], but all of them focused on the study of cementitious pores or capillary pores; macro pores were not mentioned.

From the perspective of micro size, the composition of porous concrete is mainly its macro pore structure and pore wall. The thermal and mechanical performance of porous concrete can be affected by the pore size, pore distribution, pore shape, and the connectivity of pores. Commonly used thermal conductivity models include the parallel series model, Maxwell Eucken model, effective medium theory model, Levy model, etc. These models can calculate the effective thermal conductivity of porous concrete by using the thermal conductivity of two phases in porous concrete, i.e., cement aggregate and air phase, and the corresponding volume fraction, but they could not provide information about the pore structure of porous concrete nor accurately predict the thermal conductivity of porous concrete [26].

In this paper, the AAC and the base material with different mix proportions were produced. AAC with different pore sizes but the same porosity was produced by changing the content of foam stabilizer and the stirring time of aluminum paste. The porosity and pore size of AAC were characterized by SEM testing, XRD (X-ray Diffraction) testing, and Lee’s Bottle Method test methods. The compositions of crystal were observed by XRD. The thermal conductivity of AAC and the base materials with different grades and pores were tested; then, the relationship between the porosity/thermal conductivity and the apparent density was obtained, and the relationship between the pore diameter and thermal conductivity was obtained. At last, the basic mechanic properties were tested, such as cubic strength, axial compressive strength, elastic modulus, splitting tensile strength, and flexural strength. As a series of studies, for comparative analysis, the macroscopic mechanical properties and thermal conductivity of AAC and its masonry were studied in Ref. [27].

## 2. Specimens Preparation

### 2.1. Constituent Materials

The raw materials of AAC base material—including sand, fly ash, cement, lime, gypsum, and water—the composition of AAC, and the base materials are shown in Table 1. The aluminum paste and foam stabilizer were used for AAC. Yellow River sand was used for B04 AAC and the base material, which was grinded with a cylindrical ball mill and sieved, passing a mesh of 0.075 mm with the residue of 14.3%. Quartz sand was used for B06S AAC and the base material, because quartz sand for AAC had higher purity and strength, which passed through a mesh of 0.075 mm with the residue of 18.1%.

The main function of fly ash in AAC is to provide SiO_2_ and Al_2_O_3_, which plays an important role in the static stopping process. The fly ash used in this paper was produced by Guodian Xingyang Coal Electricity Integration Co., Ltd., Zhengzhou, China. After being sieved by a mesh of 0.075 mm, the residue was 14.3%, and the ignition loss was 3.1%.

Cement is the main calcareous material in AAC. Grade 42.5 ordinary silicate cement was used in this paper; after being sieved by a mesh of 0.08 mm, the residue was 2.3%. The cement was produced by Cement Plant of China Great Wall Aluminum Co., Ltd., Zhengzhou, China.

Lime is the main calcareous material in AAC and mainly provides the effective calcium oxide. It reacted with SiO_2_ and Al_2_O_3_ in siliceous materials under hydrothermal conditions to form hydrated calcium silicate and then built the strength of AAC. At the same time, the alkaline environment due to lime hydration also provides the condition of gas generation for aluminum paste. The reaction formula is:

(1) $$Al+H_{2}O\overset{OH^{-}}{\to }Al{\left(OH\right)}_{3}+H_{2}↑$$

Lime hydration gives off a lot of heat, which provides a heat source for AAC slurry to guarantee the fully aeration of aluminum paste and raises the body temperature up to 80–90 °C during the hardening stage to promote the rapid increase of strength. However, the hydration reaction of lime increases the volume by about 44%, and the expansion of lime volume mainly occurs within 30 min after the beginning of hydration. During this period, if the control is improper, the green body with certain strength will lose plasticity, crack, and even be destroyed In this study, the material was passed through a mesh of 0.075 mm with a residual of 11.7%, the lime hydration time was 16 min with a temperature of 81.5 °C, and the effective calcium content was 72.5%.

Desulfurized gypsum used in the test was provided by Guodian Xingyang Coal Electricity Integration Co., Ltd., Zhengzhou, China. The main functions of gypsum in AAC are to regulate the aeration process by participating in the hydrothermal reaction, provide the strength, reduce the shrinkage, and improve the frost resistance of AAC.

In an alkaline environment, aluminum paste can react with water continuously to generate hydrogen until the aluminum paste runs out. In AAC, the alkaline environment is provided by Ca(OH)_2_. The reaction formula can be written as:

(2) $$2Al+3Ca{\left(OH\right)}_{2}+3CaSiO_{4}\cdot 2H_{2}O+mH_{2}O\to 3CaO\cdot Al_{2}O_{3}\cdot 3CaSiO_{4}\cdot 31H_{2}O+H_{2}↑$$

As a gas generator for AAC, aluminum paste has advantages of less dust, non-static electricity, anti-humidity, and a certain diameter role in stabilizing the pores; in comparison, dry aluminum powder produces more gas and smaller pore size. For ordinary AAC, aluminum paste was usually used in the production, and the aluminum paste used in this study was market-supplied, the solid content was 65%, the fineness was 8.94%, and the gas generation time was 17 min.

A surface-active foam stabilizer is used to reduce the surface tension of liquid water and increase the mechanical strength of the pore wall. It is the soluble oil made up of water, triethanolamine, and oleic acid in the ratio of 3.6:3:1 at room temperature. When aluminum paste releases hydrogen in slurry, the bubbles are wrapped by liquid film, and a large number of new surfaces are added to the system. Due to the extremely unstable system, the bubbles can be easily broken down; increasing the mechanical strength of the bubble film can prevent bubble rupture.

### 2.2. Preparation of Base Materials

Table 2 presents the mix proportions of one liter of AAC base materials. The dosages of sand, fly ash, cement, lime, gypsum, and water were changed to make different AAC base materials.

Since the static stop stage will directly affect the hardening degree of the green base material [28,29], three different autoclave curing systems were used for base materials to find the most suitable autoclave curing system.

The first kind of autoclave curing system adopted the same static stop and autoclave system as the AAC; that is, when the slurry had a certain strength after stopping in the grinding tool for 3 h, it was put into the autoclave for curing. The pressure in the autoclave was 1 MPa, the temperature was 185 °C, and the autoclave curing time was 9 h. The test block was found to be cracked after leaving the barrel. The reason was that the base material was different from the AAC, which had a large number of pores on the surface, and the base material was denser than the AAC. When the high-pressure steam entered the interior of the base material, the internal air flow was not smooth, and the strength of the base material was low at this time, which was not enough to resist the pressure of the steam, so it eventually led to the cracking of the base material.

The second kind of autoclave system injected the stirred slurry into the mold and then cured it for 7 days naturally. After it had a certain strength, it was put into the autoclave for curing. The pressure in the autoclave was 1 MPa, the temperature was 185 °C, and the curing time was 9 h. It was found that cracks still existed on the surface of the base material after leaving the mold, but compared with the test block made by the first autoclave curing system, the cracks of the tested block were mostly small cracks. The reason was that in addition to providing calcareous materials and an alkaline environment, the lime in the raw materials would react with water to release a lot of heat and cause the slurry expansion. Although lime was conducive to the gas generation of aluminum powder/paste and the forming of a green body, the base material did not need the gas generation; the reaction of lime and water made the internal temperature rise and caused the volume expansion of the base material in the state of natural curing, leading to a large number of small cracks in the base material before autoclave curing. Finally, it led to the cracks of the base material after leaving the mold.

In the third type of autoclave maintenance system, the lime hydration was completed by mixing with water for 30 min, and the calcium hydroxide slurry was admixed. After that, other materials were evenly mixed. The stirred slurry was injected into the mold and cured naturally for 7 days to get a certain strength for further autoclave curing in the autoclave. The pressure in the autoclave was 1.0 MPa, the temperature was 185 °C, and the curing time was 9 h. No cracks were found on the surface of the base material test block after leaving the mold.

Finally, the third base material maintenance system was adopted in this paper.

### 2.3. Preparation of AAC

Based on the mix proportion of AAC base materials, the aluminum paste and foam stabilizer were admixed for AAC. Table 3 presents the mix proportions for one liter of AAC slurry. Considering the effect of AAC density on thermal conductivity, the amount of aluminum paste was different in B05 and B06FA AAC. To ensure the pore integrity of B05 AAC, a certain amount of foam stabilizer was added. To investigate the influence of material composition on thermal conductivity, B06FA fly ash AAC and B06S quartz sand AAC were tested, respectively. In order to study the effect of pores on AAC, based on the repeated tests, for B04 AAC, the dosage of foam stabilizer was changed, and the stirring time after pouring uniform aluminum paste into the slurry was selected as 10, 20, 30, 40, and 50 s, respectively. In order to produce more gas and create a smaller pore size in AAC, dry aluminum powder was used in this study. The required aluminum paste was 48 g for 60 L B04 AAC; replacing the gas generator with dry aluminum powder, only 28 g was needed. This is due to the more than 98% content of metal aluminum and 89% content of active aluminum in dry aluminum powder compared to 70% content of metal aluminum and 85% content of active aluminum in aluminum paste [30], so the content of metal aluminum and active aluminum in aluminum powder is higher than that in aluminum paste.

In the main autoclave maintenance system of AAC, the static stopping time of slurry was about 3 h, the pressure in the autoclave was 1.0 MPa, the temperature was 185 °C, and the curing time was 9 h.

## 3. Test Methods

### 3.1. Pore Structure and Composition

XRD test: The powder samples (particle size ≤ 20 μm) of AAC and base materials were tested by Smartlab9 intelligent X-ray diffractometer of Tokyo, Japan.

SEM test: The sample size was 1 cm × 1 cm × 0.5 cm, which was measured by the FEI Inspect F50 field emission electron scanning microscope (FESEM), Portland, Oregon, USA.

### 3.2. Porosity

Apparent density and real density were measured in this test. Apparent density was measured with a ruler, the volume of the specimens was obtained, and then the mass of the specimens was accurately weighed; the apparent density was the mass divided by the volume.

The real density test steps in this paper were determined with reference to the method described in the Chinese standard GB/T208-2014 [31]. The AAC specimens were ground into powder (particle size less than 0.2 mm) and baked in an oven at 105 °C until constant weight. Then, the real densities of four kinds of AAC and base materials were measured by 25 mL of Lee’s bottle. The porosity is calculated by Equation (3).

(3) $$P=\left(1-\frac{\rho_{0}}{\rho }\right)\times 100\%$$

where *P* is porosity, *ρ* is true density, and *ρ*_0_ is apparent density.

### 3.3. Thermal Conductivity

The thermal conductivity was determined in accordance with the Chinese standard GB/T10294 [32]. The test AAC specimen size was 300 mm × 300 mm × 33 mm, and there were three test pieces in each group. The test equipment was a IM-DRY3001-I thermal conductivity tester manufactured by The Impal Measurement and Control Equipment Co., Ltd., Tianjin, China.

### 3.4. Basic Mechanical Property

**Figure 1:**
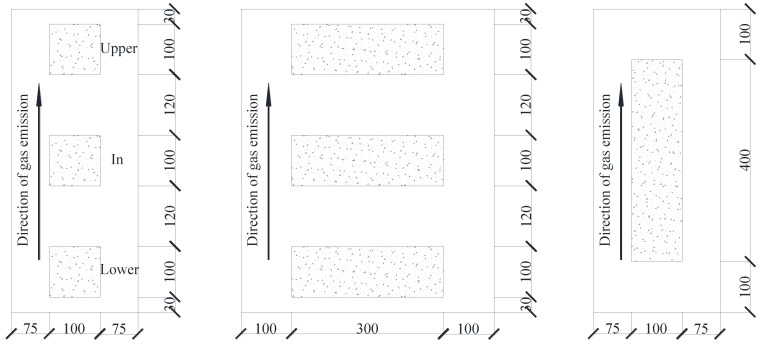
Sawing diagram of AAC specimen. (Units in mm).

The mechanical properties were tested in accordance with the Chinese standard GB11969 [33]. The base material is prepared in the molds with dimension of 100 mm × 100 mm × 100 mm, 100 mm × 100 mm × 300 mm and 100 mm × 100 mm × 400 mm. The AAC specimens were dry sawed by machine from the finished products as presented in Figure 1, and a Mj45t push table band saw produced by Jinan nasno Industrial Test System Co., Ltd. Jinan, China was used.

The blocks with dimensions of 100 mm × 100 mm × 100 mm were tested for cubic compressive strength and splitting tensile strength. Those with dimensions of 100 mm × 100 mm × 300 mm were tested for axial compressive strength and modulus of elasticity. Those with dimensions of 100 mm × 100 mm × 400 mm were tested for flexural strength. There were three test specimens in each group.

**Figure 2:**
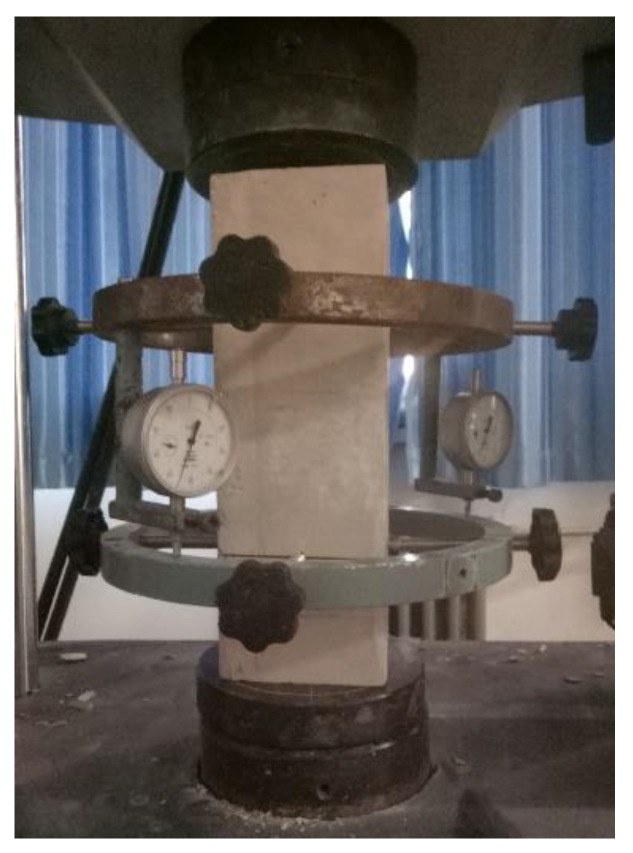
Elastic modulus test of prism.

All the mechanical tests were performed on the same machine. For the tests of elastic modulus, the micrometers with accuracy of 0.001 mm were used to measure the compressive deformation, as presented in Figure 2. The measured elastic modulus of AAC and the base material refers to the secant modulus of loading when the stress is 40% of the axial compressive strength.

**Figure 3:**
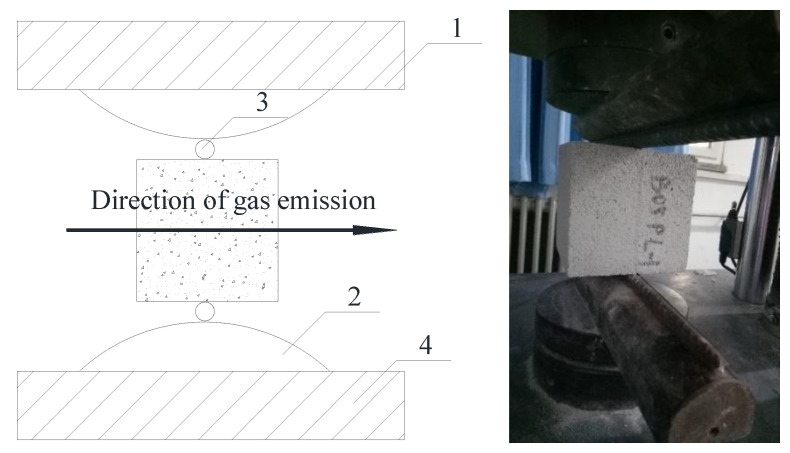
Test device of splitting tensile strength.

**Figure 4:**
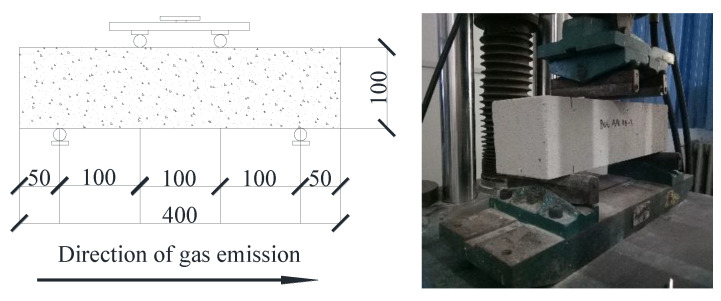
Flexural strength loading device. (Units in mm).

The splitting tensile strength test device is shown in Figure 3. The flexural strength loading device is shown in Figure 4. In both of these tests, the load was applied continuously at the speed of 0.20 ± 0.05 kN/s until the specimen was damaged.

## 4. Pore Structures

### 4.1. Compositions Observed by SEM and XRD

**Figure 5:**
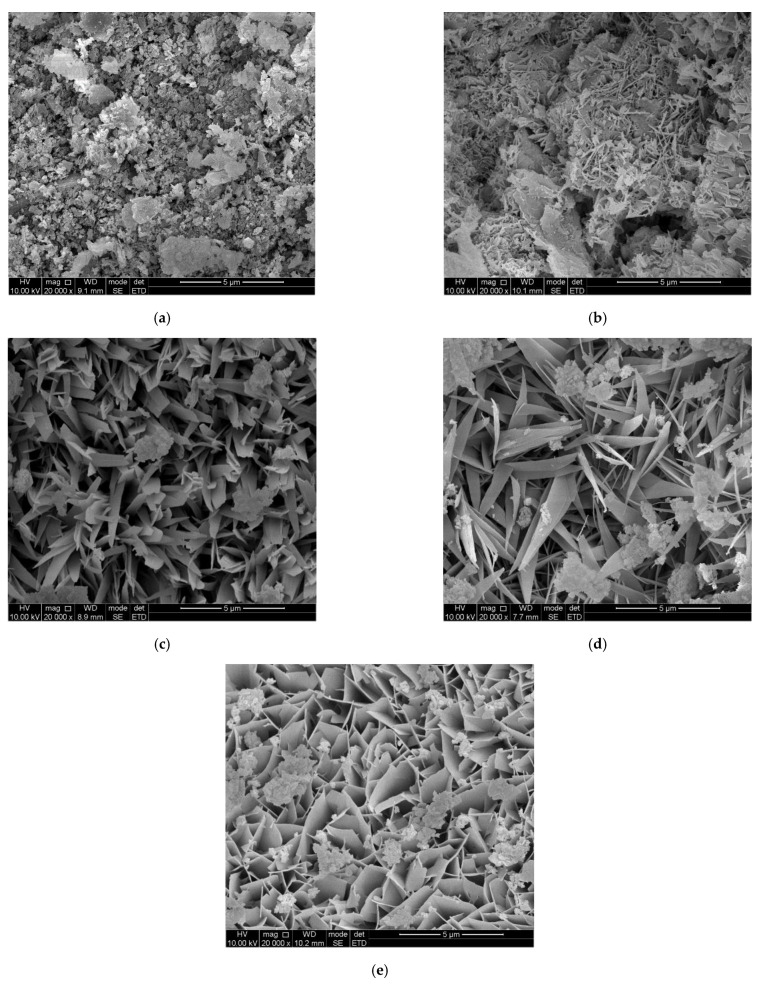
Hydration products of AAC and base material: (**a**) base material; (**b**) B04 AAC; (**c**) B05 AAC; (**d**) B06FA AAC; (**e**) B06S AAC.

The SEM images of B04, B05, B06FA, and B06S AAC are shown in Figure 5. In the figure, the form and strength of hydration products of AAC with different mix proportion are compared. It can be seen that tobermorite in B04 AAC was mainly needle-like, mainly lapped together, about 1–2 μm long, with less hydration products, more pores, and the lowest strength; tobermorite in B05 and B06FA AAC was willow-like, 1 μm wide, and 2–3 μm long, and the crystal form of the hydration product was well developed. Compared with B04 AAC, there were more hydration products in B05 AAC; the hydration products of B06FA were interlaced to form a whole body with relatively high strength. Tobermorite in B06S AAC was sheet-like and about 2 μm long, the hydration products were cross-linked with each other, forming a dense network structure with the highest mechanical strength.

**Figure 6:**
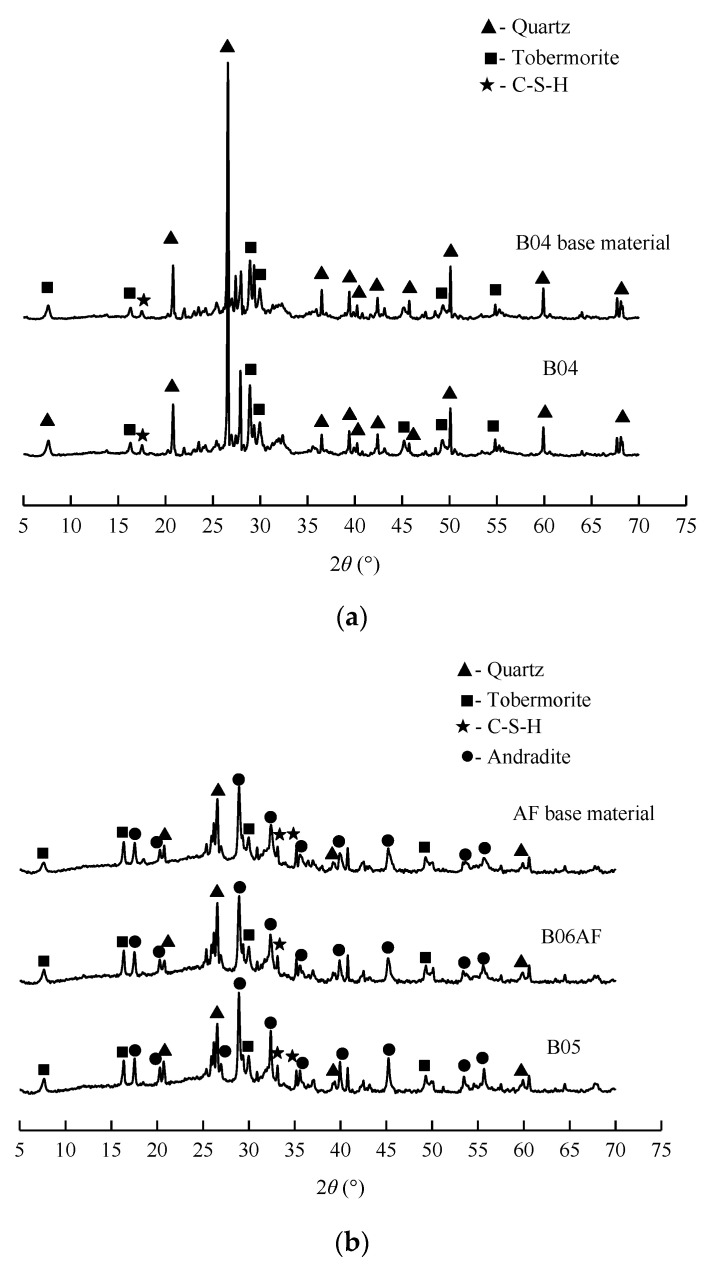

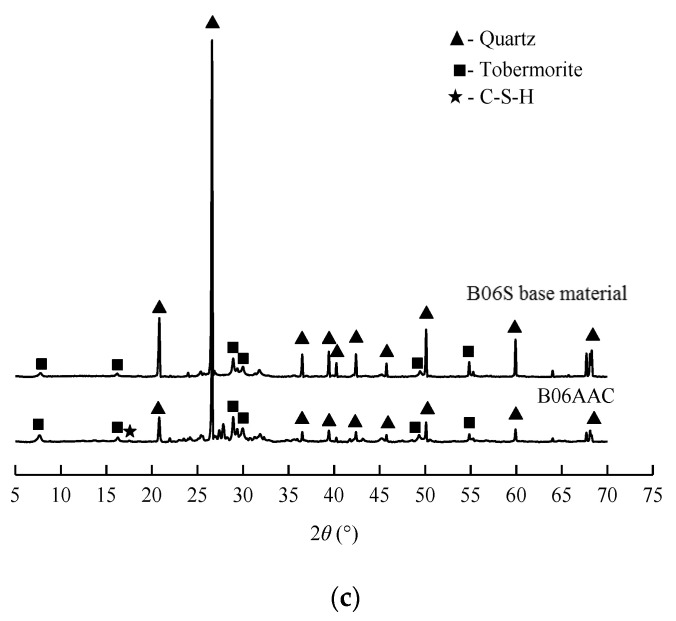
XRD (X-ray Diffraction) diffraction spectrum of AAC and base material: (**a**) B04 AAC and base material; (**b**) B05 and B06FA AAC and base material; (**c**) B06S AAC and base material.

From the XRD analysis of B04, B05, B06FA, and B06S AAC, quartz, tobermorite, hydrated garnet, and C-S-H were found in the base material, which were basically consistent with the crystals in AAC (Figure 6). Therefore, the hydration of the base material had been completed, and the autoclave maintenance system of the base material was reasonable. For four groups of AAC, the mix ratio was different, the hydration products produced were different, but the same mix ratio of the AAC and base material was almost the same. The hydration products of B04, B06S AAC, and the base material were mainly tobermorite and C-S-H gel. In addition to the hydration products, there was a lot of SiO_2_ in the crystal phase. As a result of the presence of fly ash in the mix proportion, B05, B06FA, and the FA base material were mainly composed of tobermorite, hydrated garnet, and C-S-H gel and SiO_2_; the tobermorite and hydrated garnet had higher content than the C-S-H gel.

### 4.2. Effect of Foam Stabilizer Content and Stirring Time on Pore Size

For B04 AAC, through the binarization method of AAC cross-section images, the matrix information of binary image in MATLAB was imported into Excel by using the command xlswrite(). In the Excel table, a cell represented a pixel, when the median value of the cell was 0, it represented pores, and the value of 1 represented a pore wall. The number of 0s was counted, and the porosity could be calculated. Then, VBA (Visual Basic for Applications) in Excel was used to write the macro, which could realize the one key operation when calculating the area information of a single pore. So, the pore size and quantity of the AAC surface were counted [34].

The average diameter size of each sample is presented in Table 4 and Table 5. The pore diameter is the weighted average of the pore diameter and number, which can be calculated by Equation (4).

(4) $$D_{eq}=\frac{\sum_{i=1}^{N}D_{i}}{\sum N}$$

where *D_i_* is the pore diameter, *N* is the number of the pores in 20 mm × 20 mm area section of AAC, and *D_eq_* is the average pore diameter.

**Figure 7:**
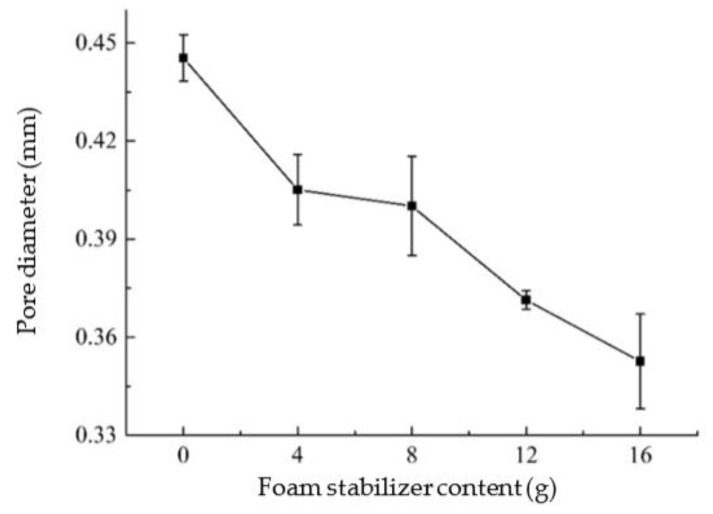
Influence of foam stabilizer content on pore diameter.

**Figure 8:**
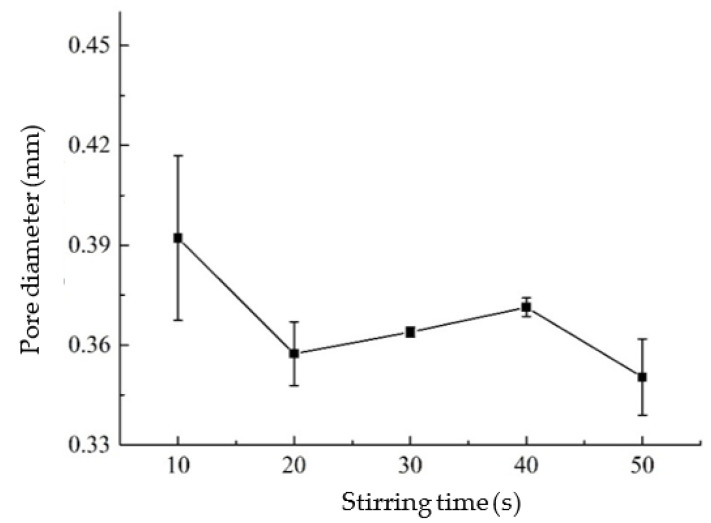
Influence of stirring time on pore diameter.

The test results show that the amount of foam stabilizer is inversely proportional to the pore diameter of AAC. This is due to the gradually expanded air foams generated by aluminum powder/paste with the increase of temperature caused by lime hydration. The foam stabilizer could alleviate the expansion of the air bubbles to a certain extent and ultimately reduce the pore size [35]. When the aluminum powder/paste was poured into the slurry, the pore size decreased with the increase of stirring time. The non-uniform presence of aluminum powder/paste in the slurry due to shorter stirring time would affect the final pore size. However, the stirring time should not be too long, nor will the aluminum powder/paste form pores after gas generation, which would lead to insufficient gas generation and increase the apparent density. From Figure 7 and Figure 8, the pore size distribution by controlling the amount of foam stabilizer is more effective than by stirring time.

From Table 5, aluminum powder instead of aluminum paste has no obvious effect on the performance of pores, but in the process of gas generation, it is found that the stirring time required for aluminum powder gas generation is shorter, and the reaction is more intense.

### 4.3. Porosity

The test results of porosity are shown in Table 6, which shows that the porosity of AAC increases with the decrease of apparent density. The base material also contains a certain amount of small pores. The porosity of AAC has exceeded 70%, and the porosity of the base material has exceeded 50%.

## 5. Thermal Conductivity Affected by Pores

### 5.1. Affected by Porosity and Apparent Density

By thermal conductivity test of the base material, the thermal conductivity of the B04 base material was 0.2358 W/(m·K), that of the B05 and B06FA base material was 0.2506 W/(m·K), and that of the B06S base material was 0.3102 W/(m·K).

The thermal conductivity and compressive strength of the AAC are presented in Table 7. With almost the same apparent density and porosity, the thermal conductivity of sand aeration AAC is 8.3% higher than that of fly ash aeration AAC, and the compressive strength is 10.4% higher, too. It is possible that the strength of sand aeration AAC is higher than that of fly ash hydration products, which leads to the higher strength of sand aeration AAC; meanwhile, the strength of sand is denser than that of fly ash hydration products, resulting in a high thermal conductivity of sand aeration AAC.

**Figure 9:**
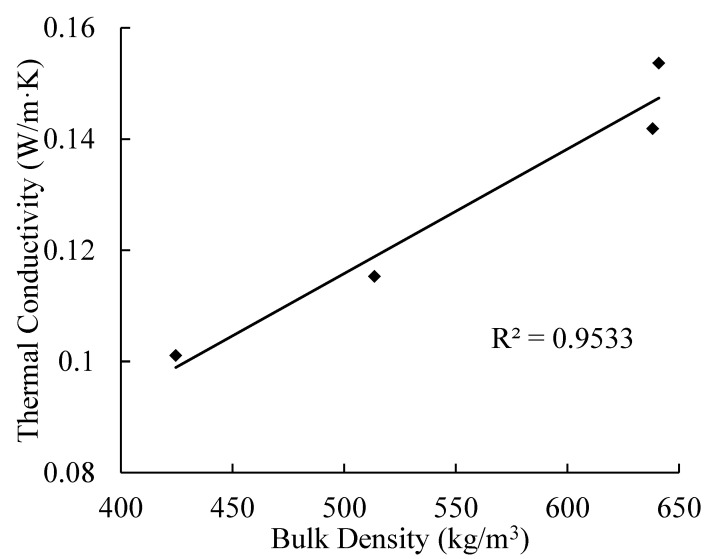
Relationship between thermal conductivity and density.

**Figure 10:**
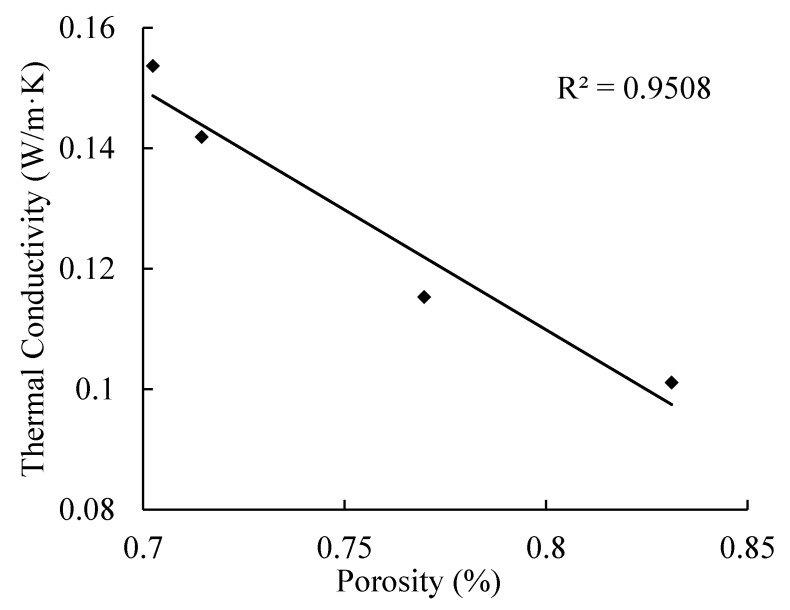
Relationship between thermal conductivity and porosity.

The relationships of thermal conductivity with apparent density and porosity of AAC are presented in Figure 9 and Figure 10. With the decrease of apparent density, the porosity of AAC increases, and the corresponding thermal conductivity decreases. By linear regression, the equations can be obtained as follows

(5) $$K_{e}=0.0002\rho +0.0039$$

(6) $$K_{e}=0.4283-0.3981P$$

where *K_e_* is the thermal conductivity.

### 5.2. Affected by Pore Diameter

For B04 AAC, the test results of the pore diameter and thermal conductivity are presented in Table 4 and Table 5. The pore diameter is the weighted average value of the pore diameter and numbers. As the volume of each test specimen is almost the same, the influence of apparent density on thermal conductivity can be neglected.

**Figure 11:**
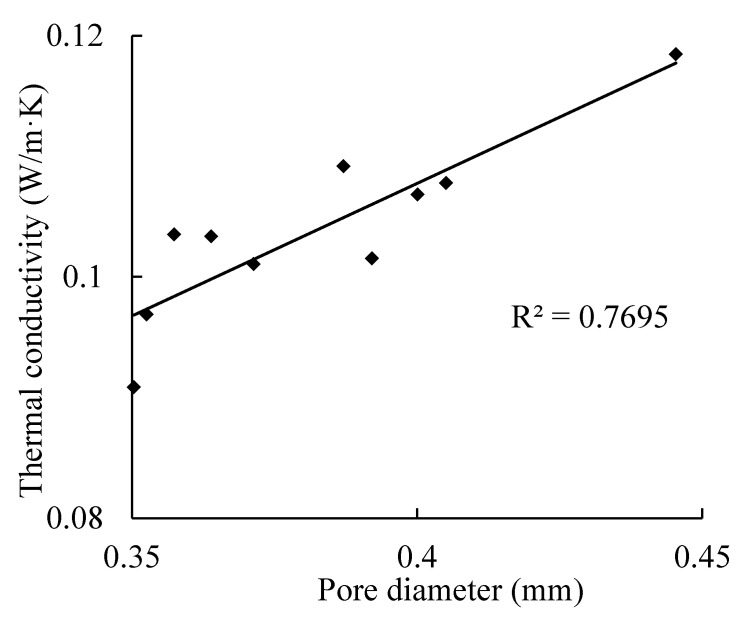
Relationship between pore diameter and thermal conductivity.

The relationship between the pore size and thermal conductivity is shown in Figure 11. The thermal conductivity of AAC increases gradually with the increase of pore size; when the pore size is 0.45 mm, the thermal conductivity increases by about 23% compared with that of 0.35 mm. This is because at the same porosity, the smaller the pore size, the more the number of pores, the path of heat transfer is increased, and the heat transfer efficiency is reduced [36]. The relation can be expressed as Equation (7).

(7) $$K_{e}=0.0198+0.2199d$$

where *d* is the pore diameter.

## 6. Cubic Compressive Strength Affected by Pores

### 6.1. Affected by Porosity and Apparent Density

For AAC, at the initial stage of loading, the specimens were in the stage of elastic loading, and there were no obvious cracks. When the load continued to increase to about 50% of the ultimate value, one or more longitudinal short cracks appeared in the longitudinal plane of the specimen. With the continuous development of the short cracks, long cracks formed, and at last, the specimen was destroyed. 

**Figure 12:**
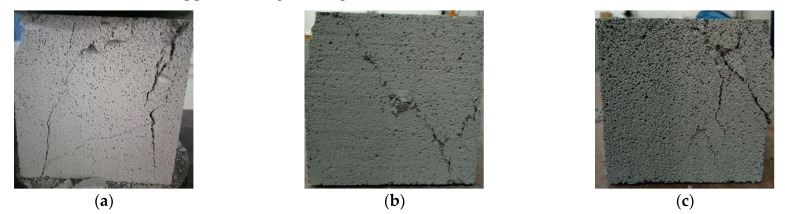
Failure mode of AAC cubic compression: (**a**) vertical crack, (**b**) oblique crack, (**c**) corner crack.

Compared with the ordinary concrete, the failure mode of AAC specimens had no obvious “X” cracks in the center. As a result of the low strength of the block, the cracks appeared irregular (Figure 12).

**Figure 13:**
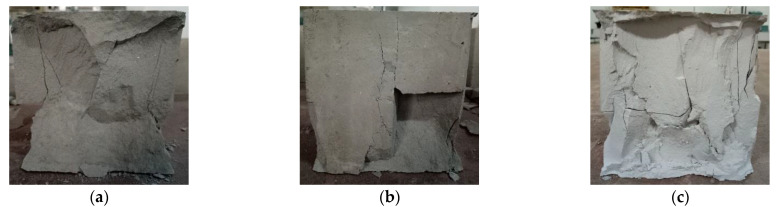
Failure mode of base material cubic compression: (**a**) “X” type crack, (**b**) corner crack, (**c**) irregular crack.

For the base material, when the base material was initially loaded, no cracks were found on the surface. With the increase of load, cracks appeared on the surface of the central part of the specimen height. As the load continued to increase, cracks developed inward. A slight splitting sound was emitted. When approaching the damage, a sound could be heard, and the specimen was damaged (Figure 13).

The failure modes of the base material were similar to those of the ordinary concrete; the central part of the crack was “X” type. Most failure modes of the base material were longitudinal splitting failure, which reflected that the lateral expansion of the block was not obvious; this may be related to the rapid release of energy inside the test block.

The test results of cubic compressive strength of AAC and base materials are shown in Table 8.

Comparing the strength and apparent density of AAC, the relationship between apparent density and compressive strength is obtained by fitting the average values of each group of data in Table 8, and the fitting result is shown in Equation (8).

(8) $$f_{cc}=0.0144\rho_{0}-4.2213$$

**Figure 14:**
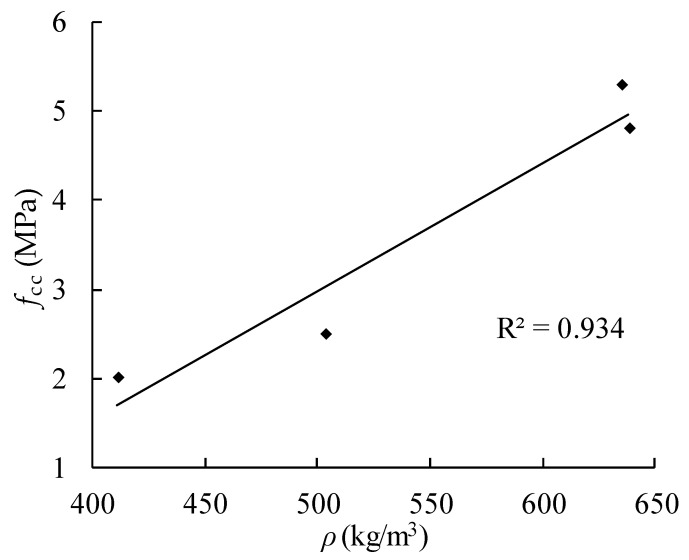
Relationship between cubic compressive strength and apparent density of AAC.

From Figure 14, the strength of AAC increases with the increase of apparent density.

From Table 8, the strength of AAC is lower than that of base materials. This is because the existence of pores greatly reduces the strength of AAC. Compared with B05 and B06FA AAC, under the same other conditions, the apparent density and cubic compressive strength decrease with the increase of aluminum paste content.

The compressive strength of AAC, compressive strength of the base material, and the porosity in Table 6 and Table 8 were fitted with the Ryshkewich formula (Equation (9)), and the result is shown in Equation (10).

(9) $$\sigma =\sigma_{0}e^{-BP}$$

where *σ* is the strength of materials with porosity *P*, MPa; *σ*_0_ is the base material strength, MPa; and *B* is empirical constant.

(10) $$f_{cc}=f_{0cc}e^{-0.08P_{1}}$$

where *f*_cc_ is the compressive strength of AAC, MPa; *f*_0cc_ is the compressive strength of the base material, MPa; *P*_1_ is the porosity difference between AAC and the base material.

Comparisons between calculated and test values of compressive strength are shown in Table 9. In the table, the average ratio of the test value to the calculated value is 1.01, and the coefficient of variation is 0.253, which shows that Equation (10) could reflect the relationship between compressive strength and porosity of AAC.

### 6.2. Affected by Pore Diameter

**Figure 15:**
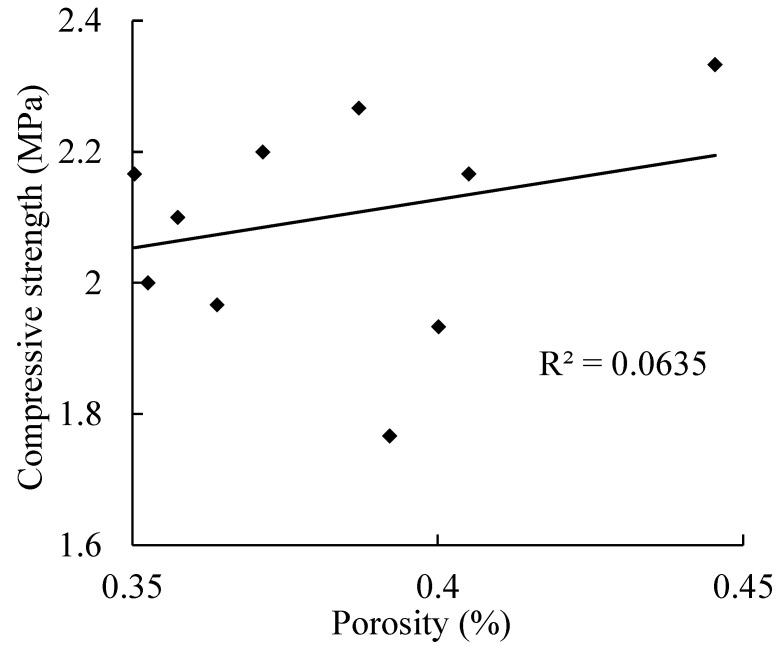
Relationship between pore diameter and cubic compressive strength of AAC.

For B04 AAC, with almost the same apparent density, the test results of pore diameter and cubic compressive strength are presented in Table 4 and Table 5, and the relationship between them is shown in Equation (11) and Figure 15. From the figure, the compressive strength of the cube increases slightly with the increase of the pore size; when the pore size is 0.45 mm, the compressive strength increases about 7% compared with that of 0.35 mm, but the increase is not so big as that of the pore size on the thermal conductivity (Figure 11), and the data discreteness is big, which shows that pore size has little effect on the compressive strength.

(11) $$f_{cc}=1.482d+1.5346$$

## 7. Basic Mechanical Properties of AAC and Base Material

### 7.1. Compressive Properties

#### 7.1.1. Axial Compressive Properties

**Figure 16:**
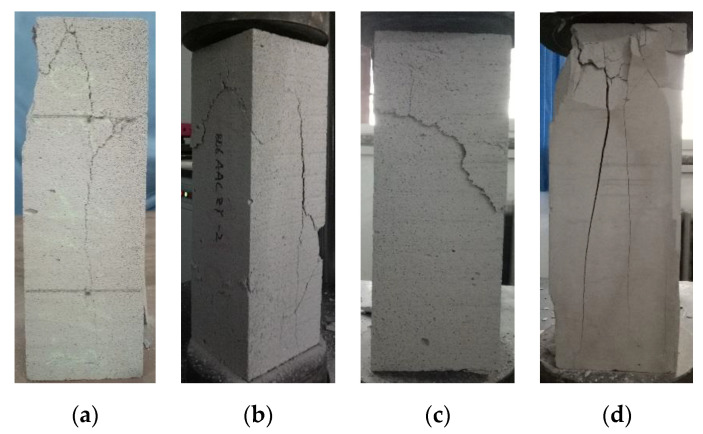
Failure mode of AAC and base material prism under axial compressive load: (**a**) AAC mode 1; (**b**) AAC mode 2; (**c**) AAC mode 3; (**d**) base material mode.

Figure 16 shows the axial compressive failure modes of AAC and the base material. The failure modes of AAC can be classified into two categories. (1) The first is longitudinal splitting failure: when the specimen was initially stressed, there was no obvious crack on the surface. With the increase of load, short cracks appeared on the surface of the specimen. With the continuous increased load, the cracks extended, and sometimes, crack branches appeared at a certain position, showing Y-type or inverted Y-type (as shown in Figure 16a,b), or there was only one main crack. (2) Due to the low strength of the specimen, internal defects, and uneven surface cutting defects, oblique cracks were formed under the load (as shown in Figure 16c). For the base material, the strength was higher than AAC, which was close to the strength of ordinary concrete, while the size of the coarse aggregate was small, and the defects between the aggregate and cement stone were small, and there were no major interface defects; once cracks appeared, the energy was released rapidly, and the specimen suddenly broke down (as shown in Figure 16d).

The results of axial compressive strength test are shown in Table 10.

In Table 10, the axial compression ratio between the axial strength and cubic strength of AAC is bigger than 0.9. For ordinary concrete, the ratio is about 0.7, the difference is due to the loose material of AAC and the limited restriction of friction resistance between the specimen and upper/lower compression plates, which makes the cubic compressive strength less improved than that of the prism. For base materials, the ratio is lower than AAC, but it is also bigger than ordinary concrete, which is because the strength of the base material is bigger than that of AAC and lower than that of ordinary concrete; the friction resistance is between the both materials.

#### 7.1.2. Elastic Modulus under Compression

The test results of elastic modulus of AAC and base material are shown in Table 11.

From Table 11, the elastic modulus of AAC and the base material increase with the increase of apparent density, and for AAC, it is smaller than that of the base material. The relationship between the elastic modulus of AAC and the axial compressive strength can be calculated by Equation (12).

(12) E=617f

where *E* is the elastic modulus of AAC, MPa; and *f* is the axial compressive strength of AAC, MPa.

#### 7.1.3. Stress–Strain Relationship

**Figure 17:**
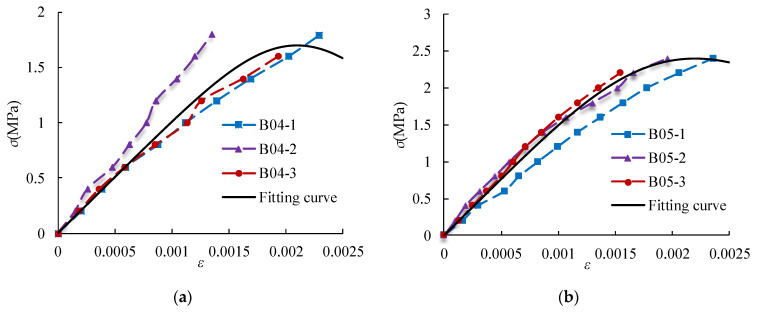

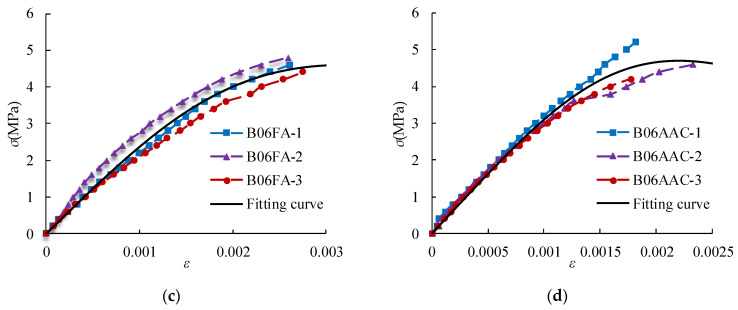
Stress–strain relationship and fitting curve of AAC: (**a**) B04; (**b**) B05; (**c**) B06FA; and (**d**) B06S.

**Figure 18:**
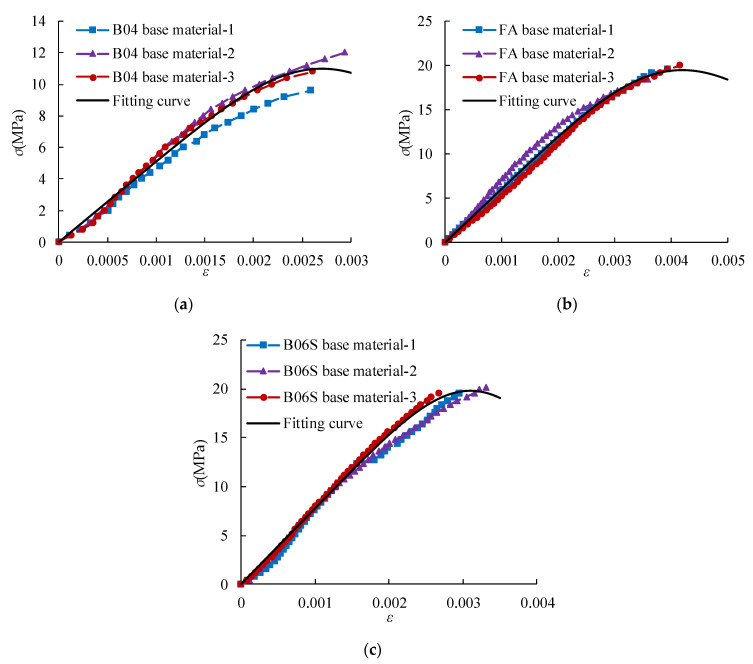
Stress–strain relationship and fitting curve of base material: (**a**) B04; (**b**) B06FA; (**c**) B06S.

The stress–strain relationship of the specimens was tested. The obtained stress–strain relationship and fitting curve are shown in Figure 17 and Figure 18.

From Figure 17, the stress–strain curves are an approximately straight line when the stress *σ* ≤ 0.4 *σ*_max_. Then, the stress increases, and the strain increases rapidly. When the stress approaches the failure load, the plastic deformation increases significantly. When the stress increases to the maximum value, the specimen is immediately damaged. For the base material, when *σ* ≥ 0.3 *σ*_max_, cracks appear on the surface of the specimen. Then, the stress increases, and the strain increases gradually. When the stress increases to the maximum value, the specimen suddenly breaks down.

The model proposed by A. Madan is used to establish the stress–strain curve relationship for AAC [37,38,39] (Equations (13) and (14)).

(13) $$\sigma =\frac{f(\frac{\varepsilon }{\varepsilon_{0}})\gamma }{\gamma -1+{\left(\frac{\varepsilon }{\varepsilon_{0}}\right)}^{\gamma }}$$

(14) $$\gamma =\frac{E_{m}}{E_{m}-E_{\sec}}$$

where *f* is the axial compressive strength (MPa), *ε*_0_ is the average peak strain (Table 11), *E*_m_ is the average elastic modulus (MPa), *E*_sec_ is the ratio of axial compressive strength to peak strain, and *γ* is a dimensionless number.

Figure 17 and Figure 18 shows that the model could well describe the stress–strain relationship of AAC and base material at the rising section of the curve.

### 7.2. Tensile Properties

**Figure 19:**
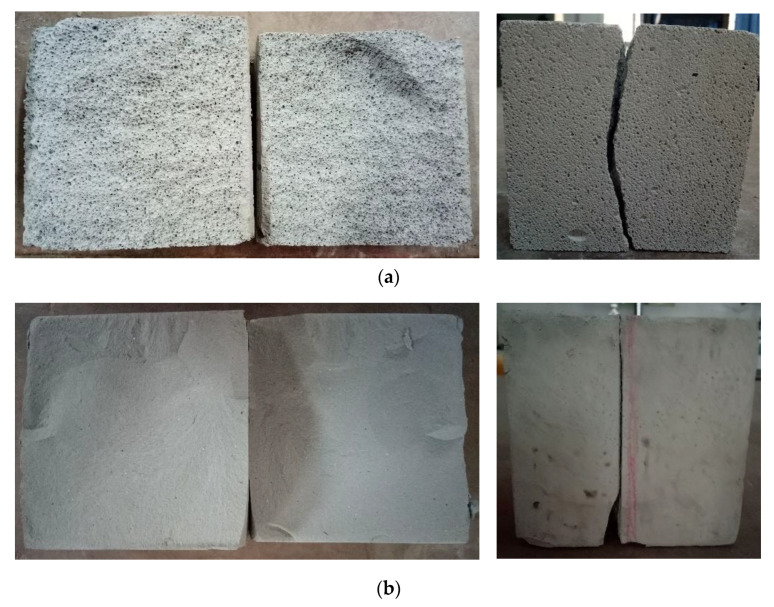
Failure pattern of splitting tensile strength: (**a**) AAC; (**b**) base material.

For AAC, there was no obvious crack on the surface of the specimens during the initial loading. With the increased load, the loading steel bars were pressed into the specimens; when approaching the limit load of the specimens, the specimens suddenly split into two halves, and the sound of brittle fracture was emitted (Figure 19a). For base material, the splitting failure is faster, and the reaction is more intense (Figure 19b).

The calculation of splitting tensile strength is shown in Equation (15). The test results are shown in Table 12.

(15) $$f_{\mathrm{ts}}=\frac{2P_{2}}{\pi A_{2}}$$

where *f*_ts_ is the splitting tensile strength, MPa; *P*_2_ is the failure load, N; *A*_2_ is the split surface area, mm^2^.

According to the analysis of the splitting tensile strength test results of AAC, the splitting tensile strength increases with the grade of AAC, and the relationship between splitting tensile strength and cubic compressive strength can be calculated by Equation (16).

(16) $$f_{\mathrm{ts}}=\alpha f_{\mathrm{cc}}{}^{0.75}$$

where *f*_ts_ is the splitting tensile strength, MPa; *α* is the regression coefficient—for AAC, *α* = 0.177, for base material, *α* = 0.132.

For ordinary concrete, the parameter *α* in Equation (16) is 0.19; for AAC, which is close to that of the ordinary concrete, but for the base material, the relationship has a bigger difference. The first reason is that the number of groups is small and the dispersion is large, while the second reason may be that the brittleness of the base material reduces the plastic fracture energy of the material [40,41].

### 7.3. Flexural Properties

When the load was applied initially, there was no obvious change in the specimen. When the maximum load was reached, the specimen suddenly broke into two parts. The test results of flexural strength are shown in Table 13. From the table, according to the formula form in Refs. [42,43], the relationship between flexural strength and cubic compressive strength can be calculated by Equation (17).

(17) $$f_{f}=\beta \sqrt{f_{\mathrm{cc}}}$$

where *f*_f_ is the flexural strength, MPa; *β* is the regression coefficient—for AAC, *β* = 0.264, for the base material, *β* = 0.298.

The data of flexural strength are fewer and more discrete; only approximate regression data are given in this paper.

## 8. Conclusions

In this paper, the preparation of AAC with different pore sizes was produced, and through many trial tests, the manufacturing method of the base material was established. Then, the related tests of the pore structure on heat transfer and mechanical properties of AAC were carried out. The conclusions could be drawn as follows:

(1) The maintenance system of the base material was determined, the stirred slurry was injected into the mold and cured naturally for 7 days to get a certain strength for further autoclave curing in the autoclave, which was a key step in the process of making the base material. From the XRD and SEM analysis of AAC, the hydration of the base material had been completed, and the autoclave maintenance system of the base material was reasonable. The properties of base material would be benefit in the meso-scale research and numerical simulation of AAC.
(2) In the test, there were two ways to change the size of the pore size in AAC: by changing the stabilizer content and stirring time. The amount of foam stabilizer was inversely proportional to the pore size of AAC. The pore size distribution by controlling the amount of foam stabilizer was more regular than that by stirring time.
(3) With almost the same apparent density and porosity, the thermal conductivity of sand aeration AAC is 8.3% higher than that of fly ash aeration AAC, and the compressive strength is 10.4% higher, too. With the decrease of apparent density, the porosity of AAC increased, and the corresponding thermal conductivity decreased.
(4) With almost the same apparent density, when the pore size was 0.45 mm, the thermal conductivity increased about 23% compared with that of 0.35 mm, but the compressive strength only increased about 7% compared with that of 0.35 mm, which means that pore size has an effect on thermal conductivity, but has it little effect on compressive strength.
(5) The basic mechanical properties of AAC and the base material were tested, and the research could provide the mechanical properties relationship between the base material and AAC. From the test results, the cubic compressive strength decreased with the increase of porosity, and the relationship between them was obtained by regression analysis; the elastic modulus was proportional to the axial compressive strength; the proposed stress–strain relationship model could well describe the relationship of AAC and base material at the rising section of curve; splitting tensile strength and flexural strength could be obtained according to the existing concrete formula.

## Figures and Tables

**Table 1:** Composition of autoclaved aerated concrete (AAC) and base materials.

Aggregate	Fe_2_O_3_ (%)	MgO (%)	CaO (%)	AL_2_O_3_ (%)	SiO_2_ (%)	SO_3_ (%)	Cl^−^ (%)	Loss on Ignition (%)
Quartz sand	0.39	0.04	0.26	0.41	99.58	--	--	--
Yellow River sand	2.58	0.08	5.94	10.61	73.81	--	--	--
Fly ash	4.82	0.76	2.58	27.6	52.99	--	--	4.64
Cement	--	2.46	--	--	--	2.54	0.018	3.09
Lime	--	2.79	75.85	--	--	--	--	--
Gypsum	85.59	--	27.87	--	--	39.81	39.81	--

**Table 2:** Mixing proportion of raw materials of AAC base material.

Specimen	Cement (g/L)	Ratio to Cement				
		Sand	Fly Ash	Plaster	Lime	Water
B04 ^1^	223.1	2.40	4.00	0.07	0.50	3.06
B05 ^1^	169.0	0	4.69	0.17	0.81	2.90
B06FA ^2^	169.0	0	4.70	0.17	0.80	2.79
B06S ^3^	161.2	5.37	0	0.18	0.85	3.15

^1^ B04, B05 represent the AAC strength level; ^2^ B06FA represents the B06 strength level and fly ash added; ^3^ B06S represents the B06 strength level and quartz sand added.

**Table 3:** Mixing proportion of raw materials of AAC.

Specimen	Cement (g/L)	Ratio to Cement					Aluminum Paste (g/L)	Foam Stabilizer (g/L)
		Sand	Fly Ash	Plaster	Lime	Water		
B04	83.3	2.40	4.00	0.07	0.50	3.06	0.8	0.2
B05	77.5	0	4.69	0.17	0.81	2.90	0.65	0.15
B06FA	92.5	0	4.70	0.17	0.80	2.79	0.50	0
B06S	83.2	5.37	0	0.18	0.85	3.15	0.50	0

**Table 4:** Relationship between pores size, thermal conductivity, and compressive strength affected by foam stabilizer.

Foam Stabilizer (g)	*ρ*_o_	*P*	Pore Diameter	Maxim Diameter	Pore Number	λ ^1^	Average	*f*_cc_^2^	Average
	(kg/m^3^)	(%)	(mm)	(mm)		(W/m·K)	(W/m·K)	(MPa)	(MPa)
	429.88	82.91	0.4412	--	--	0.1223		2.3	
0	433.84	82.75	0.4536	--	--	0.1184	0.1185	2.3	2.3
	432.66	82.80	0.4414	--	--	0.1147		2.4	
	434.88	82.71	0.4041	--	--	0.1096		2.2	
4	418.82	83.35	0.3948	2.074	743	0.1094	0.1078	2.0	2.2
	421.34	83.25	0.4164	4.425	537	0.1044		2.3	
	429.39	82.93	0.3884	--	--	0.0999		1.8	
8	426.99	83.02	0.3948	3.589	725	0.1051	0.1068	1.9	1.9
	434.64	82.72	0.4172	2.916	673	0.1155		2.1	
	428.27	82.97	0.3741	--	--	0.1010		2.5	
12	421.51	83.24	0.3685	--	--	0.1011	0.1011	2.0	2.2
	423.76	83.15	0.3715	--	--	0.1011		2.1	
	424.87	83.11	0.3591	--	--	0.0837		1.6	
16	433.87	82.75	0.3360	3.980	757	0.0999	0.0969	2.1	2.0
	434.66	82.72	0.3627	3.862	715	0.1071		2.3	

^1^ λ stands for the thermal conductivity; ^2^ *f*_cc_ stands for cubic compressive strength of AAC.

**Table 5:** Relationship between pore size, thermal conductivity, and compressive strength affected by stirring time.

Stirring Time (s)	*ρ*_o_	Porosity	Pore Diameter	Maxim Diameter	Pore Number	λ	Average	*f*_cc_	Average
	(kg/m^3^)	(%)	(mm)	(mm)		(W/m·K)	(W/m·K)	(MPa)	(MPa)
	434.64	82.72	0.3716	--	--	0.0947		1.8	
10	430.01	82.90	0.3852	4.073	651	0.1000	0.1015	1.8	1.8
	430.75	82.87	0.4196	5.483	533	0.1099		1.7	
	417.82	83.39	0.3471	--	--	0.0989		2.1	
20	422.22	83.21	0.3660	5.236	589	0.1061	0.1035	2.1	2.1
	423.64	83.16	0.3592	4.168	653	0.1056		2.1	
	422.40	83.20	0.3895	--	--	0.1088		2.1	
20 (AL ^1^)	431.10	82.86	0.4001	3.305	669	0.1110	0.1092	2.3	2.3
	432.61	82.80	0.3717	1.928	933	0.1078		2.4	
	418.20	83.37	0.3634	--	--	0.1078		2.0	
30	429.44	82.92	0.3656	3.374	780	0.1013	0.1034	1.9	2.0
	422.91	83.18	0.3627	5.308	650	0.1010		2.0	
	428.27	82.97	0.3741	--	--	0.1010		2.5	
40	421.51	83.24	0.3685	2.946	894	0.1011	0.1011	2.0	2.2
	423.76	83.15	0.3715	4.052	771	0.1011		2.1	
	424.38	83.13	0.3634	--	--	0.1015		2.4	
50	416.14	83.45	0.3420	3.649	876	0.0896	0.0909	2.3	2.2
	421.79	83.23	0.3457	5.168	769	0.0815		1.8	

^1^ AL stands for the aluminum powder instead of aluminum paste.

**Table 6:** Test results of porosity of AAC and base material.

Specimen	*ρ*_o_ (kg/m^3^)	Average (kg/m^3^)	*ρ* (kg/m^3^)	Average (kg/m^3^)	Average Porosity (%)
	393		2514		
B04	421	411	2517 ^2^	2515	83.67
	418		--		
	496		2231		
B05	513	503	2232	2231	77.47
	500		--		
	631		2236		
B06FA	640	638	2237	2236	71.66
	643		--		
	631		2154		
B06S	639	635	2154	2154	70.54
	634		--		
	1101		2377		
B04 base	1132	1108	2374	2375	53.36
	1091		--		
	1113		2509		
B05 base	1125	1127	2379	2444	53.89
	1144		--		
	1113		2509		
B06FA base ^1^	1125	1127	2379	2444	53.89
	1144		--		
	1180		2589		
B06S base	1191	1194	2587	2588	53.88
	1212		--		

^1^ The B06FA base material is the same as the B05 base material; ^2^ because the real density data are not discrete, a group of two test blocks was tested.

**Table 7:** Thermal conductivity and compressive strength of AAC.

Specimen	*ρ*_o_ (kg/m^3^)	Average (kg/m^3^)	*P* (%)	Average (%)	λ (W/m·K)	Average (W/m·K)	*f*_cc_ (MPa)	Average (MPa)
	428.27		82.97		0.1010		2.5	
B04	421.51	424.51	83.24	83.12	0.1011	0.1011	2.0	2.2
	423.76		83.15		0.1011		2.1	
	511.98		77.05		0.1146		2.9	
B05	515.02	513.56	76.92	76.98	0.1160	0.1153	3.1	3.0
	513.69		76.97		0.1153		3.0	
	640.07		71.37		0.1424		4.4	
B06FA	634.69	638.27	71.61	71.45	0.1397	0.1419	4.8	4.8
	640.06		71.37		0.1435		5.3	
	641.50		70.22		0.1523		5.5	
B06S	638.79	640.97	70.34	70.24	0.1539	0.1537	4.9	5.3
	642.62		70.17		0.1548		5.4	

**Table 8:** Test result of cubic compressive strength of AAC and base material.

Specimen	*ρ*_0_ (kg/m^3^)	Average (kg/m^3^)	*f*_cc_ (MPa)	Average (MPa)	Specimen	*ρ*_0_ (kg/m^3^)	Average (kg/m^3^)	*f*_cc_ (MPa)	Average (MPa)
	393		2.0			1101		13.7	
B04	421	411	2.0	2.0	B04 base	1132	1108	18.0	15.2
	418		2.0			1091		14.0	
	496		2.5			1113		19.5	
B05	513	503	2.5	2.5	B05 base	1125	1127	18.8	20.3
	500		2.5			1144		22.7	
	631		4.4			1113		19.5	
B06FA	640	638	4.8	4.8	B06FA base	1125	1127	18.8	20.3
	643		5.3			1144		22.7	
	631		4.9			1180		22.0	
B06S	639	635	5.5	5.3	B06S base	1191	1194	23.3	24.0
	634		5.4			1212		26.6	

**Table 9:** Comparison of calculated and tested values of compressive strength with different porosity.

Specimen	*P*_1_^1^	*f*_0cc_	Calculated *f*_cc_	Tested *f*_cc_	Calculated *f*_cc_/Test *f*_cc_
	(%)	(MPa)	(MPa)	(MPa)	
B04	30.32	15.2	1.3	2.0	0.65
B05	23.60	20.3	3.0	2.5	1.20
B06FA	17.77	20.3	4.9	4.8	1.02
B06S	16.66	24.0	6.3	5.3	1.19

^1^*P*_1_ is the porosity difference between AAC and base material, which is derived from the average value of Table 6.

**Table 10:** Test result of axial compressive strength of AAC and base material.

Specimen	*f* (MPa)	Average (MPa)	*f*_cc_ (MPa)	Average (MPa)	*f*/*f*_cc_	Specimen	*f* (MPa)	Average (MPa)	*f*_cc_ (MPa)	Average (MPa)	*f*/*f*_cc_
	1.8		2.0				11.8		1101		
B04	1.9	1.9	2.0	2.0	0.94	B04 base	11.2	10.9	1132	15.2	0.72
	1.9		2.0				9.8		1091		
	2.4		2.5				9.8		1113		
B05	2.4	2.4	2.5	2.5	0.97	B05 base	19.7	18.4	1125	20.3	0.91
	2.5		2.5				18.3		1144		
	4.0		4.4				18.9		1113		
B06FA	5.1	4.6	4.8	4.8	0.95	B06FA base	19.7	18.4	1125	20.3	0.91
	4.6		5.3				22.2		1144		
	4.3		4.9				11.8		1180		
B06S	4.7	4.8	5.5	5.3	0.90	B06S base	11.2	20.3	1191	24.0	0.85
	5.3		5.4				9.8		1212		

**Table 11:** Test results of elastic modulus of AAC and base material.

Specimen	*E* (MPa)	*E* (MPa)	*f* (MPa)	*f* (MPa)	*ε*_0_^1^	*ε*_0_^1^	Specimen	*E* (MPa)	*E* (MPa)	*f* (MPa)	*f* (MPa)	*ε*_0_	*ε*_0_
	1012		1.8		0.0023			4703		9.6		0.0026	
B04	1030	1014	1.8	1.7	0.0014	0.0019	B04 base	5301	5123	12.5	11.0	0.0032	0.0028
	1000		1.6		0.0019			5365		10.8		0.0026	
	1218		2.4		0.0024			5984		19.6		0.0039	
B05	1708	1529	2.4	2.4	0.0020	0.0021	B05 base	6871	6055	18.8	19.5	0.0038	0.0040
	1662		2.4		0.0018			5311		20.0		0.0042	
	2307		4.6		0.0026		B06FA base	5984		19.6		0.0039	
B06FA	2932	2473	4.8	4.6	0.0026	0.0027		6871	6055	18.8	19.5	0.0038	0.0040
	2180		4.4		0.0028			5311		20.0		0.0042	
	3272		5.2		0.0018			7607		19.6		0.0030	
B06S	3429	3271	4.6	4.7	0.0023	0.0020	B06S base	7939	7799	20.2	19.8	0.0033	0.0030
	3111		4.2		0.0018			7852		19.6		0.0027	

^1^*ε*_0_ is the peak strain in the stress–strain curve.

**Table 12:** Test result of splitting tensile strength of AAC and base material.

Specimen	*f*_ts_ (MPa)	Average (MPa)	Specimen	*f*_ts_ (MPa)	Average (MPa)
	0.21			0.99	
B04	0.27	0.23	B04 base	0.92	0.98
	0.21			1.03	
	0.31			1.33	
B05	0.38	0.34	B05 base	1.25	1.29
	0.34			1.29	
	0.58			1.33	
B06FA	0.58	0.60	B06FA base	1.25	1.29
	0.63			1.29	
	0.63			1.35	
B06S	0.65	0.64	B06S base	1.53	1.43
	0.64			1.42	

**Table 13:** Test result of flexural strength of AAC and the base material.

Specimen	*f*_f_ (MPa)	Average (MPa)	Specimen	*f*_f_ (MPa)	Average (MPa)
	0.33			0.21	
B04	0.34	0.36	B04 base	0.51	0.43
	0.43			0.57	
	0.34			1.74	
B05	0.19	0.26	B05 base	1.67	0.43
	0.27			1.66	
	0.36			1.74	
B06FA	0.89	0.70	B06FA base	1.67	1.69
	0.86			1.66	
	0.61			1.62	
B06S	0.56	0.61	B06S base	1.87	1.72
	0.65			1.67	

## Data Availability

Data is contained within the article.
